# Bone health in children with primary hyperoxaluria type 1 following liver and kidney transplantation

**DOI:** 10.3389/fped.2024.1353880

**Published:** 2024-02-22

**Authors:** Rainer Büscher, Lars Pape, Anja K. Büscher

**Affiliations:** Department of Pediatrics II, Pediatric Nephrology, University Hospital Essen, Essen, Germany

**Keywords:** bone health, PH1, osteopathy, children, kidney transplantation, liver transplantation

## Abstract

**Background:**

Primary hyperoxaluria type 1 is characterized by hepatic oxalate overproduction, leading to nephrocalcinosis, kidney stones, kidney failure and systemic oxalosis, including oxalate osteopathy. Combined liver-kidney transplantation (CLKT) and kidney after liver transplantation (KALT) were established therapeutic options to stop the devastating consequences of oxalate bone disease.

**Methods:**

We describe a retrospective cohort of 10 children with PH1who were referred to our hospital from different countries for combined transplantation. Demographic and clinical data were collected and symptoms of bone disease, conventional radiological examinations, plasma oxalate levels and other determinants of calcium-phosphate metabolism were compared pre and post transplantation.

**Results:**

Ten patients (7 male, median age 5.8 years, median follow-up time 8.1 years) were included in this study. Seven patients were diagnosed with infantile oxalosis and 9 patients received an intensified dialysis regime prior to transplantation. In one patient the transplanted kidney never achieved primary function and the boy remained on HD. All other patients remained without graft failure and retained stable kidney and liver function. Prior to transplantation, seven patients suffered from severe skeletal pain and three children presented with 1–3 series of pathological fractures. Pathological fractures did no longer occur in children who underwent successful CLKT or KALT. Plasma oxalate levels dropped within 6 months following Tx. Determinants of calcium-phosphorus metabolism did not differ significantly in comparison to other HD children. Seven of ten children showed a restricted growth at the time of transplantation and presented a moderate catch-up-growth at the time of last follow-up.

**Conclusions:**

Patients with PH1 suffer from severe consequences of a disturbed bone metabolism. However, bone health and growth can partially improve following CLKT/KALT.

## Introduction

Primary hyperoxaluria type 1 (PH1), a rare metabolic disorder of the hepatic glyoxylate metabolism, leads to endogenous overproduction and accumulation of oxalate in the body (1–5). Crystal deposition in the kidneys results in nephrolithiasis and nephrocalcinosis and finally causes renal failure. Once eGFR drops, oxalate crystals deposit throughout the body resulting in a multi-systemic disease and might lead to severe bone disease (5–9), anemia secondary to bone marrow replacement (6, 8), hypothyroidism (6, 8–10), retinopathy (11) and vascular disorders (6, 8). Oxalate accumulation is even more pronounced in young children with end stage renal disease (ESRD), making especially these patients at risk to develop systemic oxalosis and leading to a very high morbidity and mortality risk in this age group (3, 5, 12). Bone disease has a major impact on the patients' well-being since it leads to severe bone pain, growth retardation and bone deformities and consecutively to pathological fractures (5, 9). Therefore, early detection of this progressive disease is crucial and early treatment might improve long-term outcome.

Before the EMA and FDA approval of the oxalate production lowering agent lumasiran in 2020, liver transplantation, combined liver and kidney transplantation (CLKT) or kidney after liver transplantation (KALT) were the only established curative options to relieve patients from the devastating consequences of systemic oxalosis (13, 14). While the hepatic oxalate production normalizes after liver transplantation (LTx), it still takes several months before the body oxalate pool drops and ESRD patients who received LTx prior to renal transplantation (RTx) still require an intensified hemodialysis protocol to achieve plasma oxalate levels below a saturation level of 30 µmol/L (13). Only after RTx and normalization of kidney function, plasma oxalate levels drop. However, nobody could foresee whether correction of the body oxalate balance alone could reverse or improve already existing consequences of bone disease and how long it takes before patients achieve a normal bone metabolism. In this retrospective study, we describe a cohort of ten children and adolescents with oxalosis and severe bone disease who underwent CLKT or KALT in our center and present a follow up of the bone improvement within the years following transplantation.

### Patients and methods

Ten children and adolescents with genetically confirmed diagnosis of PH1 who received CLKT/KALT between 1998 and 2021 in the University Hospital of Essen were included in this retrospective analysis. Patients came from different European, Asian and African countries (Libya, Hungary, Sri Lanka, Greece, Israel and Germany) and were referred to our center exclusively for CLKT/KALT. They underwent more or less intensified dialysis modes prior to transplantation.

Data of all patients were collected from medical records and included patients and family history, clinical lab chemistry, genetic testing as well as additional important examinations such as abdominal/renal ultrasound. All bone Xrays were taken as needed and recorded from the Picture Archiving and Communication system at our center. Mode and duration of dialysis prior to arrival and/or transplantation at our center was also recorded when available from foreign patient's records. Clinical lab chemistry data were compared with a cohort of age-matched non PH1-patients undergoing hemodialysis in our hospital. Since all data were collected retrospectively and did not follow a specific protocol or schedule of bone health assessment, bone pain was determined unsystematically from indirect markers such as patient's history, local swelling, sensitivity to touching and local irritation. All patients' records were screened for bone specific comments or unforeseeable radiologic examinations. Follow-up data included patients and grafts outcomes and all complicatons, oxalosis and non-oxalosis related. The glomerular filtration rate (GFR) has been estimated according to the Schwartz formula. Post-transplant catch-up growth is presented as change in body height Standard Deviation Score (SDS).

This study was approved by the ethics committee at the University of Duisburg-Essen [protocol number 15-6259-BO] and all patients gave their written consent to publish health related data and pictures taken for medical purposes.

Statistical analysis was performed using GraphPad Prism© (version 5.01 for Windows, GraphPad Software, San Diego, California, USA). Normally distributed data are presented as mean ± standard deviation and non-normally distributed data as median and range. A *p*-value of <0.05 was considered significant.

## Results

Data from 10 children have been included in this study (7 male, median age 5.8 years, range 0.4–17.8 years) with a median follow-up time of 8.1 years (range 3–17 years). *AGXT*-gene mutations have been reported in ten children: 7 patients with homozygous *AGXT*-gene mutations, one patient with 2 heterozygous mutations, and 2 patients with not further differentiated *AGXT*-gene mutations (Table 1). Seven children were diagnosed with infantile oxalosis. None of our patients received lumasiran at any time. Four children received CKLT (mean age at CKLT 12.6 ± 3.9 years) and 6 KALT (mean age at LTx 1.4 ± 0.9 years and RTx 2.2 ± 1.1 years; Table 1). In 4 patients split liver segments were from living related donors and all other livers were from deceased donors. Nine children underwent an intensified hemodialysis regimen with 5–6 HD sessions for 4–6 h/day per week prior to RTx and daily peritoneal dialysis treatment. One kidney was living donated and 9 kidneys were from deceased donors. In one patient, the transplanted kidney did not show primary function and the boy remained on HD for the following 3 months until renal graft function normalized. Another patient never showed primary kidney function after transplantation and the boy (patient 10, Table 1) is still on hemodialysis. All other patients remained without graft failure and retained stable kidney and liver function with observation times exceeding 10 years (Table 1).

**Table 1:** Outcomes of CLKT/KALT in 10 patients with confirmed diagnosis of PH1.

#	*AGXT*-Gene Mutation	Infantile oxalosis	Initial mode of dialysis	Age at CLKT (years)	Liver survival (months)	Kidney survival (months)	Patient survival (months)/Time of last follow up	Bone pain /fractures prior/post-Tx
1	Not further differentiated	−	−	17.8	84	84	84	−
2	Exon 4; homozygous p.Gly170Arg	+	PD/HD	11.8	195	195	195	+/fractures
3	Exon 4; homozygous p.Gly170Arg	−	PD/HD	8.1	139	139	139	+/fractures
4	Not further differentiated	−	HD	12.6	145	145	145	−
**#**				Age at KALT (years)	Liver survival (months)	Kidney survival (months)	Patient survival (months)	
5	Exon 4; p.Gly170Arg and Exon 8; p.Ile279Thr	+	PD/HD	Liver 1.7; kidney 3.1	>169	>152	>169	+
6	Exon 4; homozygous p.Gly170Arg	+	PD/HD	Liver 1.9; kidney 2.4	>75	>68	>75	+
7	Exon 4; homozygous p.Gly170Arg	+	PD/HD	Liver 0.4 kidney 1.4	>100	>88	>100	(+)
8	Homozygous p.Ile244Thr	+	PD/HD	Liver 0.7; kidney 1.1	>180	>168	>180	(+)
9	Homozygous c.956C > T	+	PD/HD	Liver 0.8; kidney 1.4	>104	>96	>104	(+)
10	Exon 10; homozygous pVal324Glyfs*7	+	PD/HD	Liver 2.9; kidney 3.9	>36	HD, no primary function	>36	+/fractures

PD Peritoneal dialysis; HD, Hemodialysis; + = patient suffers from severe bone pain (e.g. swelling, sensitive to touch, local irritation); (+) severe bone pain is not directly described.

**Figure 1:**
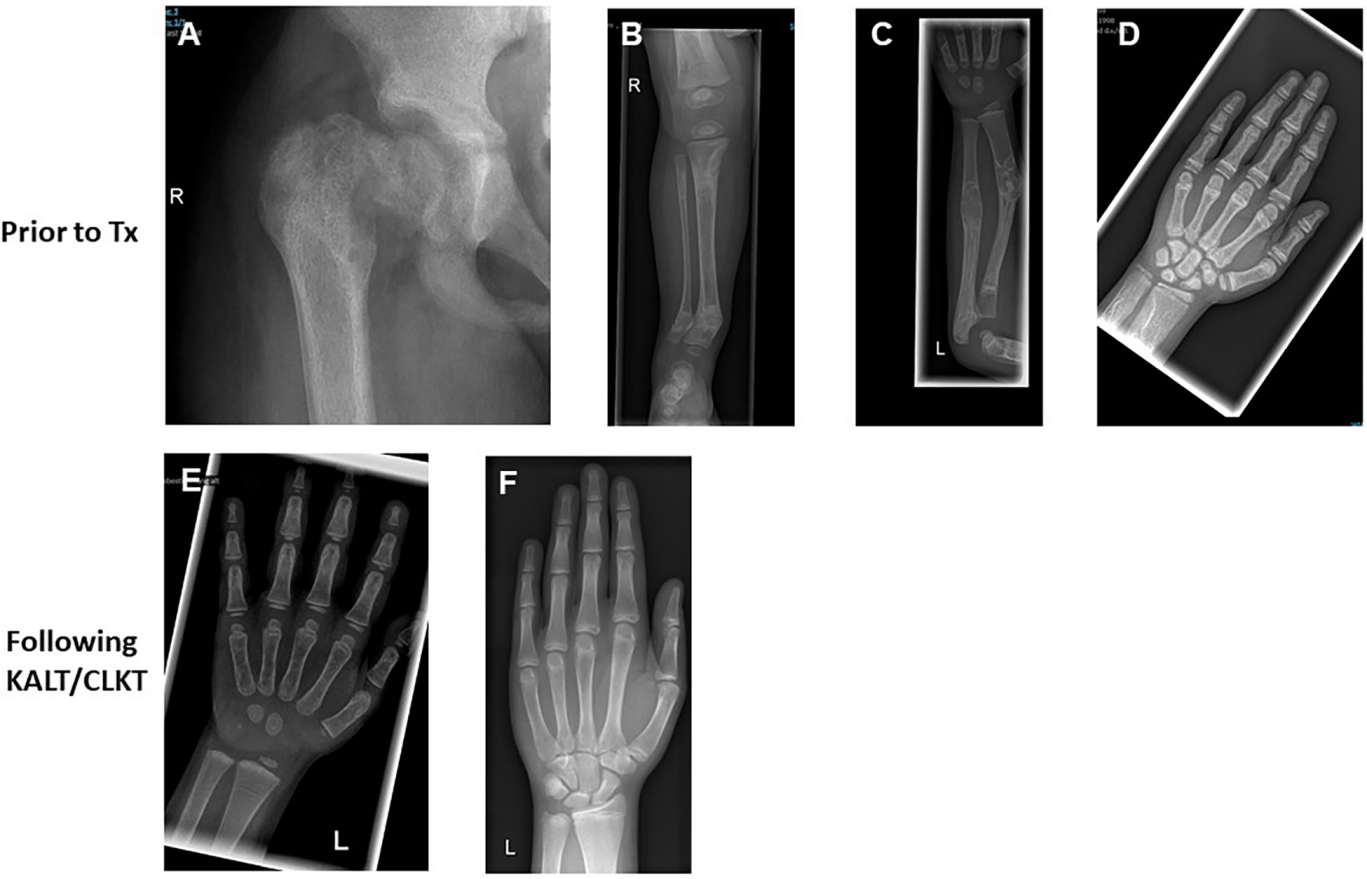
Skeletal changes in patients with PH1 prior and post CLKT and KALT. (**A**) ♀, 10 years, HD + PD; reduced bone calcification; osteopenic diaphysis; femoral head slipped to caudal (e.g., pathologic fracture of the femoral neck; older fatigue fractures). (**B**) ♂, 2 years, HD, uncompleted fracture of the right medial tibia in an area of increased sclerosis. (**C**) ♂, 5 years, HD, condition after KALT (no kidney function); constant dystrophy and increased sclerosis of radius and ulna with zigzag of the bone; several older fractures. (**D**) ♀, 11 years, HD + PD; increasing spotted decalcification of the sceletal system. (**E**) ♀, 2.7 years, condition after KALT 12 months before; calcification only slightly decreased. (**F**) ♂, 16 years, condition after CLKT; only slight bone sclerosis; no signs of renal osteopathy.

All 10 patients had various radiological signs of osteodystrophy. Five patients suffered from severe skeletal pain and 3 children presented with 1–3 series of pathological fractures of the hip, femoral neck or upper arm prior to transplantation (Table 1 and Figure 1). Furthermore, two patients were unable to walk and needed a wheelchair.

Plasma oxalate levels dropped from 115 ± 40 µmol/L to 12 ± 8 µmol/L within 6 months following Tx (Table 2). When stratified by mode of transplantation (Table 3), children undergoing CLKT tend to show lower plasma oxalate levels 6 and 12 months after transplantation when compared to children undergoing KALT (6 ± 2 vs.17 ± 12 µmol/L after 6 months). However, this fails to reach statistical significance (*p* = 0.11). Determinants of calcium-phosphorus metabolism (PTH, 25-hydroxy vitamine D, calcium, phosphorus and alkaline phosphatase) did not differ significantly between PH1 patients and other children treated with HD following Tx and normalized after renal transplantation (Table 2). Seven of ten children showed a restricted growth at the time of transplantation (−1.1 ± 0.4 SDS) and presented a moderate catch-up-growth at the time of last follow-up (−0.8 ± 0.2 SDS).

**Table 2:** Time course of eGFR, plasma oxalate, blood chemistry and SDS height of patients with PH1.

	Prior to CLKT/KALT (*n* = 10)	6 months (*n* = 10)	12 months (*n* = 10)	18 months (*n* = 10)	24 months (*n* = 10)	30 months (*n* = 10)
eGFR (ml/min/1.73 m^2^)	Treated with dialysis	81 ± 12	75 ± 12	83 ± 9	80 ± 6	80 ± 6
Bone pain (*n*)	7	5	3	2	–	–
Height (SDS)	−1.1 ± 0.4	−1.1 ± 0.5	−1.1 ± 0.3	−0.9 ± 0.4	−0.8 ± 0.3	−0.8 ± 0.2
Plasma oxalate (µmol/L)	115 ± 40	12 ± 10	11 ± 8	nm	nm	nm
PTH (pg/ml)	258 ± 12	160 ± 110	55 ± 15	65 ± 15	60 ± 14	82 ± 1
25-hydroxy-vitamine D (ng/ml)	28 ± 10	30 ± 8	22 ± 4	25 ± 12	24 ± 7	29 ± 6
Calcium (mmol/L)	2.3 ± 0.2	2.0 ± 0.4	2.0 ± 0.2	2.1 ± 0.2	2.1 ± 0.2	2.1 ± 0.4
Phosphorus (mmol/L)	3.4 ± 2.2	2.1 ± 0.9	1.8 ± 0.2	1.8 ± 0.8	1.9 ± 1.1	1.5 ± 0.3
AP (U/L)	538 ± 210	236 ± 73	229 ± 58	199 ± 48	210 ± 30	214 ± 15

Nm, not measured; eGFR, estimated glomerular filtration rate; AP, alkaline phosphatase; PTH, Parathyroid hormone; SDS, Standard deviation score.

**Table 3:** Plasma oxalate levels (µmol/L) prior and post transplantation, stratified by transplantation mode (CLKT or KALT).

Patient # (CLKT)	Prior to transplantation (*n* = 4)	6 months (*n* = 4)	12 months (*n* = 4)
1	116	7	6
2	156	8	7
3	187	5	5
4	89	4	5
Mean ± SD	137 ± 43	6 ± 2	6 ± 1
Patient # (KALT)	Prior to transplantation (*n* = 6)	6 months (*n* = 6)	12 months (*n* = 6)
5	145	20	18
6	89	12	14
7	89	25	30
8	89	8	12
9	56	3	8
10	130	35	7
Mean ± SD	98 ± 30	17 ± 12	15 ± 8

## Discussion

Among the three known types of primary hyperoxaluria, PH1—PH3, infantile PH1 is the most common and aggressive one which shows rapid progression to ESRD within the first few years of life (12, 15). Before new RNA interference-based therapeutic options (RNAi) became available in 2020, which target the reduction of liver oxalate production (16), CLKT or KALT was preferentially performed in patients with severe forms of PH1 who did not benefit from or respond to vitamin B6 therapy (13, 14). Although liver transplantation corrects the underlying metabolic disorder, the harm by the already existing body oxalate pool is devastating and besides renal failure, patients suffer extremely from bone disease and skeletal disorders (5–7, 9). While several studies describe the bone morphological and skeletal changes in patients with PH1 (5–7, 9), only few reports focus on the long term follow up of patients who successfully underwent CLKT or KALT (13, 15, 17). We have previously reported a favorable surgical outcome in pediatric PH1 patients undergoing CLKT or KALT (13) and provide new evidence that these patients partially recover from bone disease. Especially, bone pain disappeared in all patients within 18 months following Tx and they showed a moderate catch-up growth and bone re-calcification. Plasma oxalate levels dropped under the saturation level within 6 months and further electrolyte disturbances were not different from other patients who underwent HD and renal transplantation for other reasons. Our results are in good agreement with other studies showing that patients show at least a moderate catch-up-growth within the observation period (17).

Our paper tries to address two important facts of PH1 bone disease: First, it is a description of the current subjective and objective bone markers of a rare metabolic disorder, such as occurrence of bone pain, amount and quality of pathological fractures and disturbances of the electrolyte and vitamin-D-related metabolism. Pathological fractures occurred in all age groups and seemed to be independent from the length of ESRD or time spent on dialysis. However, the changes were more predominant in young children who did not receive an intensified HD protocol from the beginning, e.g., patient # 10, who came as fugitive and had received CAPD instead of HD for a longer period. We have recently demonstrated that only an intensified HD protocol is capable to reduce plasma oxalate levels significantly (1) and might therefore reduce the effects caused by the disturbed bone metabolism (12, 18). However, there is no clear evidence in the literature whether severity of bone disease is linked to individual plasma oxalate levels or the accumulated body oxalate pool (6, 15). Secondly, and this is new and important in this retrospective analysis, many changes were at least partially reversible regardless of the age of onset and we describe an approximate time course following CLKT and KALT. Although patients will probably never gain a normal catch-up-growth or complete bone repair, our study might add important information to provide a quantitative severity score in the future. However, it is very challenging to diagnose bone metabolism and link the outcome to certain metabolic parameters (18). Analysis of the previous few pediatric descriptions are important and necessary, but are more of historic value and have to be reevaluated in the near future when long-term studies of PH1 patients undergoing newer therapeutic strategies are available (12, 15–18). So far, it remains only speculative whether new therapeutics have the potential to fully replace liver transplantation in the future. In our opinion, this is highly dependent on the already existing individual damage of systemic oxalosis, the age of onset of renal failure and probably the underlying genotype. We are currently treating a one year old girl from Sri Lanka who was diagnosed with infantile oxalosis at the age of 2 months. The patient carries a very pathogenic loss of function mutation in Exon 1 of the *AGXT* gene (p.Lys21Glnfs*156), receives an intensified HD and PD treatment and is treated with lumasiran from her third months of life. She is now one year old and only poorly responding to the new therapy with oxalate levels remaining very high. In this case, it is hard to imagine that only renal transplantation is sufficient to improve the girl's outcome.

Our study has several limitations, primarily caused by its small size and descriptive and retrospective character. Furthermore, our study has a selection bias since we mainly report patients who were exclusively transferred to our center for the option of CLKT or KALT and underwent more or less intensified dialysis protocols in their countries of origin. Unfortunately, we did not follow a specific protocol or schedule of bone health assessment in our study and bone pain was determined unsystematically and not assessed qualitatively from indirect markers such as patient's history, local swelling, sensitivity to touching and local irritation. However, all patients’ records were screened for bone specific comments or unforeseeable radiologic examinations. The skeletal status pre and post-transplantaton was derived from Xray analysis only when needed and bone mineral density was not measured (5). The epidemiology of bone impairment is difficult to assess, and it remains unclear whether certain levels of plasma oxalate or urine oxalate excretion are linked to the severity of bone disease. Xrays are only indirect, surrogate markers and do not allow exact interpretation of the bone mineralization or level of osteodystrophy. However, osteosclerosis, decalcification and calcification prior and post transplantation can be assessed satisfactorily by conventional Xrays and might therefore be sufficient to evaluate the stability of the bone.

In conclusion, patients diagnosed with PH1 suffer from severe consequences of systemic oxalosis and show a disturbed bone metabolism. Following successful CLKT or KALT, changes of bone morphology are partially reversible and patients suffer less from bone pain and show at least a moderate catch-up growth within a couple of years. Future prospective studies are necessary to evaluate whether modern therapies like lumasiran make liver transplantation or CLKT obsolete and have an impact on the severity of bone disease.

## Data Availability

The original contributions presented in the study are included in the article/Supplementary Material, further inquiries can be directed to the corresponding author.
